# *Telipogon peruvianus* (Orchidaceae) Flowers Elicit Pre-Mating Behaviour in *Eudejeania* (Tachinidae) Males for Pollination

**DOI:** 10.1371/journal.pone.0165896

**Published:** 2016-11-03

**Authors:** Carlos Martel, Lianka Cairampoma, Fred W. Stauffer, Manfred Ayasse

**Affiliations:** 1 Institute of Evolutionary Ecology and Conservation Genomics, Ulm University, Helmholtzstraße 10–1 Containerstadt, D-89081, Ulm, Germany; 2 Institut für Spezielle Botanik und Botanischer Garten, Johannes Gutenberg Universität, D-55099, Mainz, Germany; 3 Conservatoire et Jardin Botaniques de la Ville de Genève, Université de Genève, CP 60, Chambésy, 1292, Geneva, Switzerland; Ciudad Universitaria, ARGENTINA

## Abstract

Several neotropical orchid genera have been proposed as being sexually deceptive; however, this has been carefully tested in only a few cases. The genus *Telipogon* has long been assumed to be pollinated by male tachinid flies during pseudocopulatory events but no detailed confirmatory reports are available. Here, we have used an array of methods to elucidate the pollination mechanism in *Telipogon peruvianus*. The species presents flowers that have a mean floral longevity of 33 days and that are self-compatible, although spontaneous self-pollination does not occur. The flowers attract males of four tachinid species but only the males of an undescribed *Eudejeania* (*Eudejeania* aff. *browni;* Tachinidae) species are specific pollinators. Males visit the flowers during the first few hours of the day and the pollination success is very high (42% in one patch) compared with other sexually deceptive species. Female-seeking males are attracted to the flowers but do not attempt copulation with the flowers, as is usually described in sexually deceptive species. Nevertheless, morphological analysis and behavioural tests have shown an imperfect mimicry between flowers and females suggesting that the attractant stimulus is not based only on visual cues, as long thought. Challenging previous conclusions, our chemical analysis has confirmed that flowers of *Telipogon* release volatile compounds; however, the role of these volatiles in pollinator behaviour remains to be established. Pollinator behaviour and histological analyses indicate that *Telipogon* flowers possess scent-producing structures throughout the corolla. Our study provides the first confirmed case of (i) a sexually deceptive species in the Onciidinae, (ii) pollination by pre-copulatory behaviour and (iii) pollination by sexual deception involving tachinid flies.

## Introduction

Animal-pollinated plants have evolved various floral signals in order to attract their pollinators, which are usually rewarded with pollen and nectar while visiting and pollinating flowers [1–3]; however, certain plant species deceive their pollinators by using attractive floral signals that mimic signals that play a role in food-seeking behaviour and the reproductive biology of their cheated pollinators [4–6]. Among the pollination-cheating systems, a remarkable example is sexual deception that, except for a few cases [7–8], is almost exclusively found in the large monocotyledonous family Orchidaceae, in which it has evolved independently in several phylogenetically non-closely related groups [6, 9–11]. Sexually deceptive orchids predominantly attract hymenopteran males for pollination [12–22], although males of Diptera and Coleoptera have also been reported [15, 20, 23–25]. Sexually-aroused males usually show copulatory behaviour with the flower (so-called pseudocopulation); during these processes, the pollinia are attached to the male insect bodies and are later transferred to the stigma of another flower, thereby pollinating it [12, 26–29]. Three key factors indicate the occurrence of sexual deception in a pollination system (for details see criteria in [24]): (a) only adult males act as pollinators; (b) the pollinators develop pre-copulatory or copulatory behaviour on the flower; (c) only one or two pollinator species are usually involved in the syndrome (see [15, 24–25, 30]).

Sexually deceptive orchids have been documented in Asia, Australia, South Africa, South and Central America and Europe [11, 25, 31]. Most of the sexually deceptive orchid species have, to date, been studied in Australia and Europe (for reviews [5, 11, 15, 32]), In spite of their high diversity, neotropical orchids have been neglected. Although requiring much further work to confirm, Tropical America may yet prove to contain the highest number of sexually deceptive plants species in the world. For example, more than 800 species have been described in a single genus *Lepanthes*, which is suspected to be entirely sexually deceptive [23]. Van der Pijl and Dodson [33] proposed several cases of sexual deception for the Neotropics, including the genera *Telipogon* and *Trichoceros*. However, confirmed sexually deceptive taxa have only been reported in the genera *Bipinnula* (as *Geoblasta*; [19]), *Lepanthes* [23], *Mormolyca* [18] and *Trigonidium* [17].

*Telipogon*, together with the genera *Hofmeisterella* and *Trichoceros*, forms the *Telipogon* alliance [34]. Deception seems to be the rule in the alliance, since no rewards are available for floral visitors [35]. The alliance is considered to be sexually deceptive based on early reports of Dodson [36] and van der Pijl and Dodson [33]. They have recorded tachinid males as being attracted by flowers of *Trichoceros antennifer* (as *Tr*. *parviflora*) but, unfortunately, their reports are contradictory. Initially, Dodson [35] pointed out that the pollinaria of *Tr*. *antennifer* became attached to the legs of flies, whereas van der Pijl and Dodson [33] described the pollinaria as being attached to the abdomen. Anecdotal reports have suggested two mechanisms for pollination in *Telipogon*: (a) flowers mimicking tachinid females to attract males (sexual deception; [33, 35–40]) and (b) flowers mimicking prey items to attract host-seeking females [41]. However, other than anecdotal observations, detailed records documenting pollination events in the *Telipogon* alliance are so far absent.

The genus *Telipogon* occurs in Central to South America but most species are found in mid-elevations of the Andean cloud forests, coinciding with the highest diversity areas of tachinids [42], their presumed pollinator taxa. Many *Telipogon* species are characterized by having the appearance of an insect sitting at the centre of the flower and their flowers commonly bear spiny calli and columns resembling, at least to human eyes, the spiny abdomens of tachinid flies [35, 38–39, 41]. Although all flowers are known to produce volatiles, *Telipogon* flowers have been recorded as ‘scentless’, as they are notable for their lack of detectable scent to humans, but no chemical analysis of the group has been reported; therefore, visual stimulus is believed to be of major importance to attract pollinators [35, 38–39, 43]. However, some species (e.g. *Telipogon peruvianus* T.Hashim) bear bald columns lacking a developed callus and, therefore, the "classic" visual stimuli are not present.

Tachinid flies are generalist pollinators; they are abundant and conspicuous nectarophagous flies [44], and several species can be found on flowers of a single plant species [44–45]. Some tachinids show male aggregations on hilltops (‘hilltoping’; [46]), where perched males wait for the arrival of receptive females or fly from landmark to landmark without reference to any topographical feature [47]. Little is known about the mating behaviour of tachinids and only a few species have been studied [48].

The aim of our investigation was to clarify the mechanism of pollination in *Te*. *peruvianus*. We have tried to answer the following questions. (a) Do tachinid flies pollinate *Te*. *peruvianus* flowers and do these flowers morphologically mimic the females of their pollinators? (b) Do the males display pseudocopulatory behaviour? (c) Are the flowers scented in this taxon and, if so, what classes of compounds are present in their odour bouquet? Using various methodological approaches, we show that sexual deception indeed occurs in *Telipogon* orchids and that a specific male tachinid is the pollinator.

## Materials and Methods

### Plant species

**Fig 1:**
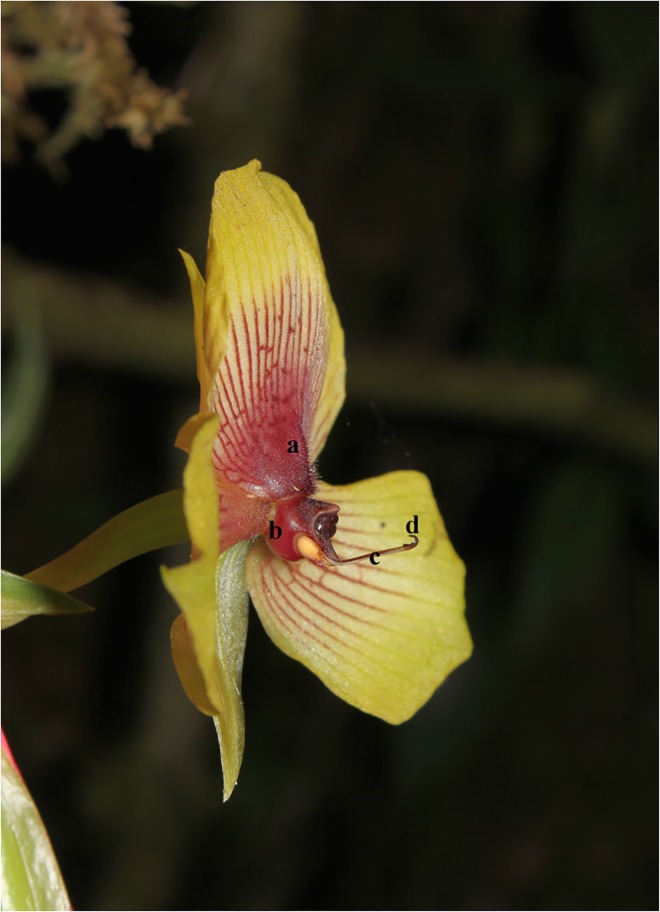
*Telipogon peruvianus* flower. Side view of the flower showing the central flower parts: (a) reduced callus, (b) column, (c) stipe and (d) the hook-like viscidium. The diameter of the flower is approximately 5 cm. Photograph by M. Ayasse.

*Telipogon peruvianus* is an epiphytic perennial herb with a highly restricted distribution in the Araza and Q’eros basins at an altitude of between 2600 and 3000 m in Cusco, Peru. It grows in semi-dense populations and several plants can often be found in one single tree. It is commonly present in semi-disturbed areas, along trails, near to light gaps or at forest boundaries. The flowering season is between June and September. The flowers of *Te*. *peruvianus* bear bald columns and lack characteristic setae or a conspicuous callus on the labellum (Fig 1). The flowers of this species possess a pollinarium with two pairs of differently sized pollinia, a long stipe and a hook-like viscidium (Fig 1; [49]).

### Study site

Field studies were carried out during the flowering seasons between 2012 and 2016 in the cloud forest of the Araza river basin near Marcapata town, southeast Peruvian Andes at an altitude of 2800–3000 m (13°35’32.04”S 70°58’32.19”W). Observations were made on individuals of two nearby patches (P_1_ & P_2_) at a distance of 350 m from each other. P_1_ was located in a forest on the foothills of the mountains and the plants of *Te*. *peruvianus* were exposed to direct sunlight conditions for several hours per day, whereas P_2_ could be found in a patch of semi-plain forest and the plants of *Te*. *peruvianus* were exposed to direct sunlight conditions only during the late morning and early afternoon. Fieldwork was carried out with the permission of the Dirección General Forestal y de Fauna Silvestre (Peruvian Ministry of Agriculture and Irrigation).

### Flower anthesis and breeding system

Flower longevity was determined by labelling flowers (n = 12) and plants (n = 12) with flagging tape before flower anthesis (bud phase) followed by daily checking until the flowers withered. The flowers were recorded at full anthesis when the column was totally exposed. The end of anthesis was considered to be reached when the labellum and petals began to withdraw. Flower changes during anthesis were recorded. To ensure no pollination during the complete floral anthesis, adhesive tape was used to cover the stigma. Flowers were not bagged to avoid exposing them to any extra moisture that could harm them.

To measure the pollination success in *Te*. *peruvianus*, we assessed the quantity of the pollinated flowers (in the form of pollen deposition or fruit formation) in all of the observed flowers (n = 85 and n = 60 for P_1_ and P_2_, respectively) and counted the number of pollinated plants (in the same way) in all of the observed plants (n = 39 and n = 48 for P_1_ and P_2_, respectively).

To determine the species breeding system (self-fertilization or xenogamy), we performed three treatments: (a) non-manipulated flowers (control, n = 12) to examine spontaneous self-pollination and agamospermy; (b) flowers self-pollinated by hand and hand cross-pollinated flowers of the same plant to examine self-compatibility (n = 17); (c) hand cross-pollinated flowers of different plants (n = 10) to examine cross-pollination. For hand-pollinated treatments, complete pollinaria from diverse plants from the two patches were collected by using a metal tweezers; only one pollinium was directly transferred to each stigma of the treated flowers. Treated stigmas were protected with adhesive tape as explained above.

### Flower-visiting behaviour and frequency of pollinators

All the observations were carried out in the two previously mentioned patches (P_1_ and P_2_) with, in total, 180 h of observations (120 h in P_1_, 60 h in P_2_). Daily observations were made between 8:00 and 16:00 h. The behaviour of flower visitors and frequency of pollinator visits were recorded for 15 min per hour (e.g. 8:15–8:30 h; 9:15–9:30 h and so on) but only when the day was sunny, as tachinid activity is strongly dependent on weather conditions ([33], Martel pers. obs.). The behaviour of male pollinators was classified and quantified into stages: a) inspection (seeking behaviour at a distance between 1 m to 20 cm from the flower); b) approaching (seeking behaviour shown at a distance of less than 20 cm from the flower); c) touching (touching the centre of the flower with the legs); d) landing (landing in the centre of the flower); e) pseudocopulation (copulation attempt with the centre of the flower). A flower visit was considered when a male touched, landed or pseudocopulated with the flower. The pollination process was recorded by means of photographic and video devices. Pollinators, with or without attached pollinaria, and floral visitors were captured by means of insect-nets while or after visiting the flowers or during nectar feeding on neighbouring plants in order to identify species and sex identity.

### Comparison of male behaviour at females and flowers

To identify whether pollinator behaviour on a flower was similar to that shown in the presence of female visual and tactile stimuli, an odourless female dummy (Soxhlet extracted pollinator female) was pinned on a leaf. Below the leaf an intact *Telipogon* flower was placed, which was hidden from and invisible to male visitors. The odourless dummy provided the same visual and tactile stimuli than an alive female and the flower the olfactory stimuli. This setup was then offered to pollinators and their responses were recorded during 15 min. The recorded responses per trial were then transformed to a percentage (total number of inspections, approachings, touchings and landings equal 100%) and compared with the responses of the pollinators in the presence of flowers.

### Morphology and comparative anatomy

Floral diameter was measured by means of a digital calliper, the length of the labellum plus the petal being measured. In the field, floral buds, open flowers, male pollinators and females of the pollinators were collected, fixed and stored in a solution of 70% ethanol. In the laboratory, we analysed whether the structure (macro- and micro-morphology) of the central region of the flowers morphologically mimicked the body (pilosity, hairs and cell morphology) of the female of the pollinator. The column and callus of the flowers and the abdomen and thorax of the flies were measured. Stereomicroscopic analyses were performed with a Carl Zeiss stereomicroscope (Stemi 2000-CS) coupled with a digital camera (Canon EOS 500D). Scanning electron microscopy (SEM) analyses were carried out on female flies and flower structures (lip, petals and column) of *Te*. *peruvianus* in order to compare their micro-structures. Samples for SEM analyses were dissected, dehydrated, critical-point dried and sputter-coated with gold for viewing in a Hitachi S-5200 in-lens Scanning Electron Microscope at an accelerating voltage of 5 kV at the Central Facility for Electron Microscopy of Ulm University.

### Scent-producing organs and light microscopic analyses

We used neutral red staining and light microscopy to identify potential areas of scent emission on the labellum and petals of flowers. Fresh flowers and flower pieces were stained in a solution of neutral red-water (diluted 1:10,000) [50]. Neutral red is a weak cationic dye that indicates cell permeability, which is correlated with the presence of glands, such as scent-producing structures; however, it can also stain other kind of flower structures such as nectaries. For light microscopy, labella and petals of mature buds were collected and fixed in 70% ethanol. They were then infiltrated and embedded in Technovit 7100 (2-hydro-xyethyl methacrylate). Serial sectioning was carried out on a rotary microtome (Microm HM-355) to produce sections of 5–6 μm in thickness. This work was carried out at the Laboratory of Plant Systematics and Biodiversity of the Conservatory and Botanical Garden of Geneva. All sections were stained with ruthenium red and toluidine blue and permanently mounted in Histomount. Observations were performed on a Carl Zeiss (Axio Scope.A1) microscope coupled to a digital microscope camera (AxioCam ICc3). Micrographs were processed with Axiovision Rel. 4.8 software (Carl Zeiss).

### Flower scent collection and chemical analyses

In preliminary investigations Headspace samples of flowers, using filters with adsorbents (for methodological details see [51]), were collected. This technique is well known to be effective for detecting highly volatile compounds that are emitted in large amounts into the headspace [52]. However, solvent extracts are preferred for the detection of volatiles emitted in trace amounts and for less volatile compounds. As prior collected Headspace samples showed substances in traces amounts only, extracts were preferred. For extraction, four flowers of *Te*. *peruvianus* were cut off from plants of P_2_ and their labella were washed in 4 ml pentane (99.9%, HPLC grade, Sigma-Aldrich) for 24 h. Samples were analysed by using gas chromatography (GC) and a gas chromatograph-mass spectrometer (GC-MS). GC was equipped with an unpolar DB5 capillary column (30 m × 0.25 mm i.d. J&W) and a flame ionization detector (FID). GC-MS was performed with a double-focusing VG70/250 SE mass spectrometer (Vacuum Generators Ltd.) linked to an HP 5890 gas chromatograph (Hewlett-Packard) equipped with an unpolar DB5 capillary column (30 m × 0.25 mm i.d. J&W) and a mass selective detector (MSD). Hydrogen and helium were used as the carrier gas for GC and GC-MS, respectively. Aliquots of 1 μl sample were injected splitless at an oven temperature of 50°C. After 1 min, the splitter valve was opened and the temperature was increased at a rate of 8°C/min to 310°C. Structure elucidation of individual compounds was performed by comparing the mass spectra in our samples with those of commercial libraries (NIST library, ADAMS, Library of the Institute of Evolutionary Ecology and Conservation Genomics, Ulm University) and with the spectra of synthetic compounds. Double-bond positions in unsaturated hydrocarbons were assigned according to Buser et al. [53] and Dunkelblum et al. [54]. The relative proportions of identified compounds were assessed based on peak areas.

## Results

### Flower anthesis and mating system

*Telipogon peruvianus* flowers remain at anthesis for roughly one month (mean ± SD: 33 ± 9.47 days, n = 12) ranging from 21 to 51 days. Flowers (n = 12) need one to three days to open completely. During floral aging, flower colouration changes from dark-red to pale-brown. Up to two flowers at a time can be found in an inflorescence (mean ± SD: 1.89 ± 1.05 flowers, n = 40) and up to two flowering branches per plant have been recorded (mean ± SD: 1.13 ± 0.37 branches, n = 40).

The pollination success varied greatly between the two patches. In P_1_, 42% of the flowers were pollinated (n = 85) and 56% of the plants presented fruits (n = 39) and in P_2_, 3.33% of the flowers were pollinated (n = 60) from two plants producing fruits (4.17%, n = 48). No spontaneous self-pollination was recorded in any flower. All the hand-pollinated flowers exhibited fruit development (n = 27), although whether the fruits produced more seeds with one treatment or another was not quantified. When pollinated, the stigma took about five days to dissolve the pollinium, with flowers withering after 7 days following pollination (mean ± SD: 7.08 ± 0.51 days, n = 12).

### Pollination process, pollinator behaviour and pollinator frequency

**Fig 2:**
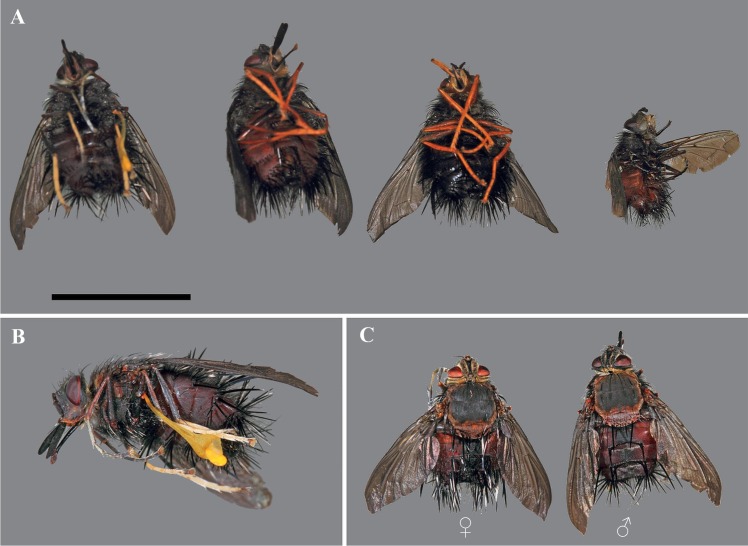
Tachinid collected attracted by *Telipogon peruvianus* flowers. (A) Males of diverse tachinid species (from left to right: *Eudejeania* aff. *browni*, *Eudejeania subalpina*, *Eudejeania* sp., *Peleteria* sp.) attracted by the flowers of *Telipogon peruvianus*. (B) Male of *Eudejeania* aff. *browni* with a pollinarium attached to its leg. (C) Dorsal view of a male and female of *Eudejeania* aff. *browni*. Photographs by H. Bellman.

Only four fly species, all male tachinids and never females, were attracted to flowers of *Te*. *peruvianus*: *Eudejeania* aff. *browni* (an undescribed *Eudejeania* species; M Wood, pers. comm.), *Eudejeania* sp., *Eudejeania subalpina* Townsend and *Peleteria* sp. (Fig 2). Nevertheless, *Eudejeania* sp. (n = 3) and *E*. *subalpina* (n = 12) never landed on flowers but some insects did closely approach the flowers to within less than 5 cm. Although *E*. aff. *browni* and *Peleteria* sp. were observed to touch or land on flowers, only the former was observed to carry pollinaria (n = 15, *E*. aff. *browni* males carrying pollinaria). Furthermore, *Eudejeania* aff. *browni* was the most common tachinid fly visiting the flowers (n = 55), whereas males of *Peleteria* sp. were observed on only six occasions (during the whole observation time span). Thus, *E*. aff. *browni* flies were significantly more attracted and performed more visits to these flowers than *Peleteria* sp. flies (binomial test: p < 0.001). *Peleteria* sp. males never pollinated flowers and were much smaller than the *E*. aff. *browni* males (*E*. aff. *browni* size [min-max]: 18–20 mm, n = 5; *Peleteria* sp. size [min-max]: 12–15 mm, n = 3; Fig 2).

Flies of *E*. aff. *browni* were observed visiting *Te*. *peruvianus* (n = 55) in P_1_ but none was recorded in P_2_. Male and female flies look similar in colour and morphology, with females exhibiting slightly bigger abdomen. However, the two sexes are easily recognized in flight, with males normally performing very fast movements when moving from one flower to another, whereas females clearly fly more slowly and appear less shy than males (Martel pers. obs.). The flies after visiting flowers and on the surrounding nectar plants were found to be exclusively *Eudejeania* aff. *browni* males. (n = 25); 15 amongst them had pollinaria attached to their legs.

**Fig 3:**
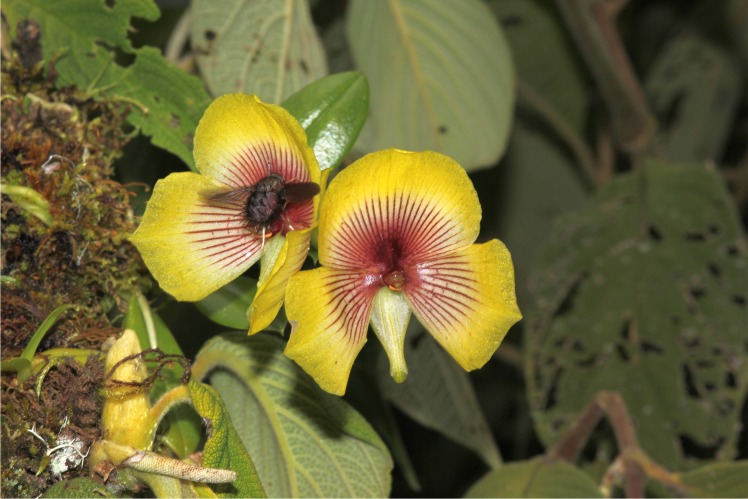
*Eudejeania* aff. *browni* male visiting a *Telipogon peruvianus* flower. The male is performing pre-copulatory behaviour on a flower. Note the legs grasping the flower even before the male has landed. Photograph by M. Ayasse.

All the male tachinids approached the flowers with fast movements and showed characteristic flying movements similar to the movements expected for an insect following an odour plume. While approaching the flowers, the males of *E*. aff. *browni* flew in circles around the flowers (once or twice) and afterwards either flew away from the flower or immediately approached its central region (see S1 Video). When males closely approached the flowers, they started touching the column, the petal and the labellum base with their legs (Fig 3). They then flew away or landed and continued touching the central region of the flower by performing fast leg movements. During these movements, the femur of their legs made contact with the viscidium and then the pollinarium became attached to the flies (>30 pollination events observed). After landing, males usually left the flowers rapidly with the pollinarium (always <5 seconds after landing; n = 26). The process was highly effective, since we only recorded flowers (n = 36) with one pollinium in each visited stigma; on the other hand, some observed males (n = 6) carried more than one pollinarium (up to 7 pollinaria). Anther caps were not observed on pollinaria attached to fly legs or on stigmas. Thus, the caps were lost after pollinarium removal and before pollinium deposition, probably by friction when the males dragged the caps during walking on leaves, flowers and soil.

**Fig 4:**
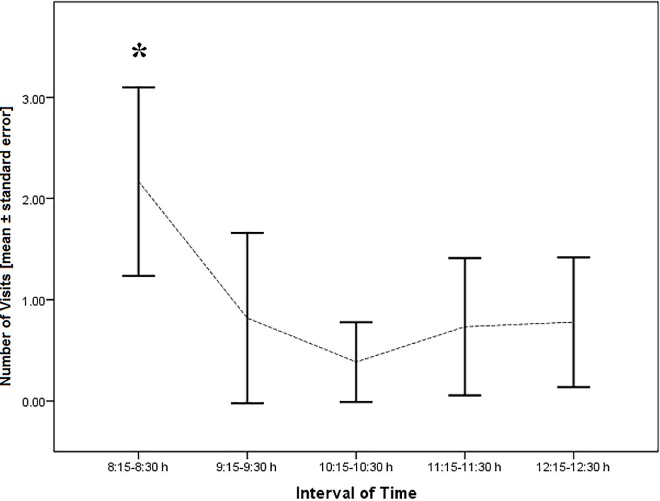
Frequency of pollinator visits to *Telipogon peruvianus* flowers. The number of male pollinators observed during the first interval of time (8:15–8:30 h) was significantly higher (*) than during the other intervals (Mann-Whitney-U test with Benjamini-Hochberg correction, p<0.05). Bars denote error and the dotted line the pattern of visits along the observation time.

In P_1_, *Telipogon peruvianus* flowers were observed to be visited by males of *E*. aff *browni* between 8:00 and 13:00 h; however, the main frequency of visits was registered during the first interval (mean ± SD: 1.67 ± 1.41 visits) soon after the sunlight directly irradiated the study site. The frequency of visits was significantly smaller in the subsequent time intervals (Mann-Whitney-U test with Benjamini-Hochberg correction, p<0.05): during the next interval, the frequency of visits dropped (mean ± SD: 0.78 ± 1.30 visits) and remained constant until 13:00 h (Fig 4). No visits were recorded after 13:00 h. Furthermore, in the study area, the weather usually became cloudy after midday.

Dozens of males of *E*. aff. *browni* were observed patrolling a transect of ca. 200 m length which presented various nectar-producing non-orchid plants such as *Baccharis* sp. (Asteraceae), *Dendrophorbium longilinguae* (Asteraceae), *Rubus roseus* (Rosaceae) and *Symplocos melanochroa* (Symplocaceae), although they seemed to prefer feeding on flowers of *R*. *roseus* and *D*. *longilinguae*. Females of *E*. aff. *browni* were also observed on flowers of *R*. *roseus* and *D*. *longilinguae*. Nine females in total were caught around nectar-rewarding plants but none of them presented with a pollinarium attached or were observed to approach *Te*. *peruvianus* flowers.

### Comparison of male behaviour at females and flowers

**Fig 5:**
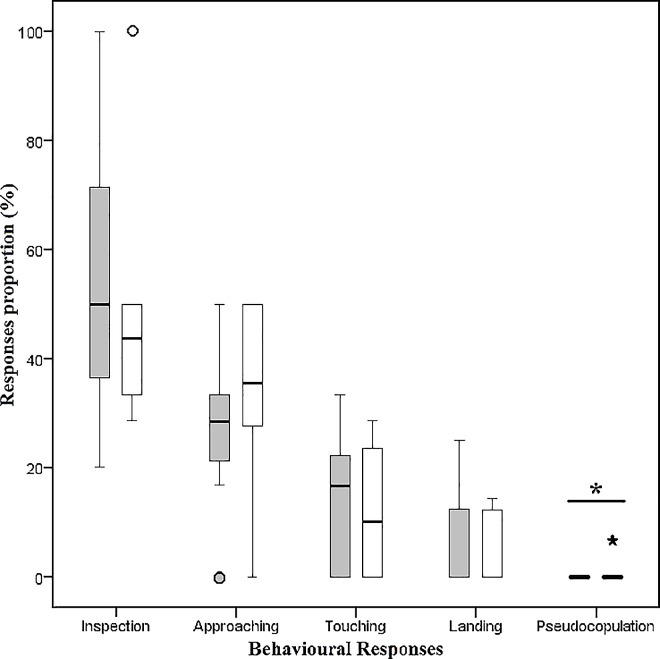
Behavioural responses of *Eudejeania* aff. *browni* males to flowers of *Telipogon peruvianus* and female dummies. The proportion (in percentage) of behavioural responses (i.e. inspection, approaching, touching, landing and pseudocopulation) of males in the presence of flowers (grey boxes) and female dummies (white boxes) was not significantly different, except for pseudocopulation (*) (Mann-Whitney-U test, p<0.05). Bars denote error.

The pattern of behavioural responses of *E*. aff. *browni* males in the presence of flowers (n = 37) and dummies (n = 8) was similar (Fig 5, Table 1). Although no pseudocopulatory behaviour was recorded in any of the males visiting a flower, 0.8% of the recorded behavioural responses for a male visiting a dummy were pseudocopulations. The male after touching the body of the dummy, landed and moved behind the female; this is consistent with the behaviour observed on flowers, except for the pseudocopulatory response. No significant differences were observed for any of the behavioural responses of males to flowers and dummies (Table 1; Mann-Whitney-U test, p>0.05), except for pseudocopulation (Mann-Whitney-U test, p = 0.032) (Fig 5).

**Table 1:** Behavioural responses (in percentage; mean ± SD) of *Eudejeania* aff. *browni* males in presence of *Telipogon peruvianus* flowers (n = 37) and female dummies (n = 8).

	Inspection	Approaching	Touching	Landing	Pseudo-copulation
**Flower**	50.5 ± 29.6	22.8 ± 15.3	12.2 ± 11.4	4.8 ± 8.2	0.0 ± 0.0
**Dummy**	48.1 ± 22.6	34.8 ± 16.9	12.2 ± 13.2	5.0 ± 6.9	0.8 ± 2.4

### Comparative anatomy

**Fig 6:**
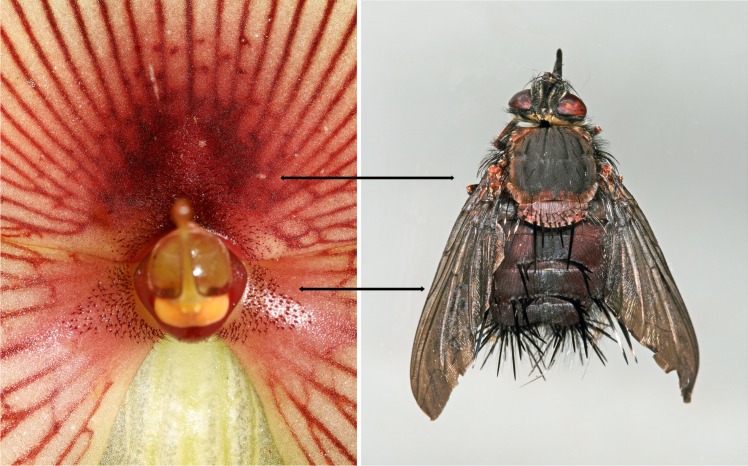
Comparative morphology of *Telipogon peruvianus* flower (mimic) and the pollinator’s female (model). Arrows show the mimicked areas (callus and column) and their models (thorax and abdomen). Photographs by M Ayasse (left) and H Bellmann (right).

Flowers of *Te*. *peruvianus* are non-resupinate. They measure about 6 cm (mean ± SD: 5.98 ± 0.86 cm, n = 35) in diameter, the petals and labellum being similar in length but the labellum being wider than the petals. Petal margins are bright yellow, followed by a white area having dark red veins leading to the centre at which a red spot can be found. The labellum bottom presents a reduced callus (dark red area) with dark red trichomes (Fig 6). Trichomes are also present on the base of petals.

Male and female tachinids of *E*. aff. *browni* appear to humans eyes to be morphologically similar and only differ in abdominal length and width, although some overlap has been observed in body size (Fig 2). The abdomen bears long thick black bristles. The thorax is hairy (except for the scutellum) with small black bristles (Fig 6). The morphological analyses of *Te*. *peruvianus* suggest that the callus mimics the thorax and that the column mimics the abdomen of the female flies (Fig 6). The thorax and abdomen of the female flies are considerably larger than the callus and column, respectively.

**Fig 7:**
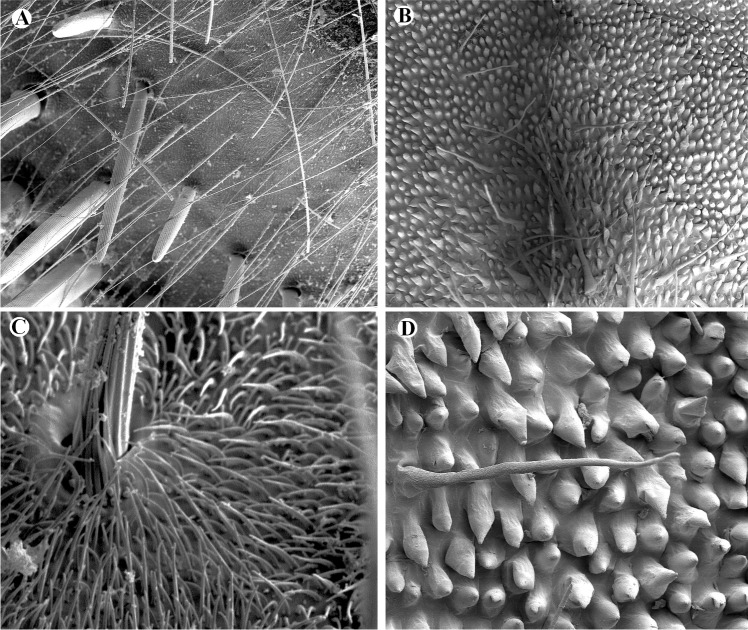
SEM micrographs of *Telipogon peruvianus* flower and *Eudejeania* aff. *browni* female. (A) Thorax of a *Eudejeania* aff. *browni* female and its bristle types. (B) Reduced callus of *Telipogon peruvianus* and its trichomes and conical epidermal cells. (C) Details of the short and large bristles of the thorax. (D) Details of a trichome and conical epidermal cells of the callus. Photographs by C Martel.

SEM-based analyses of female flies and flowers showed limited morphological mimicry with respect to pilosity (Fig 7). The callus is covered with conical and elongated papillate cells (min-max: 15–25 and 40–60 μm long, respectively) on the adaxial epidermis surface. The reduced callus also presents unicellular bristle-like trichomes (min-max: 0.25–1.1 mm long). The dorsal part of the column has a flat surface, with slight grooves on the cell walls, but no trichomes were observed. Males and females of *E*. aff. *browni* present three kinds of bristles: (a) small bristles densely covering the entire abdomen and thorax surface (min-max: 10–15 μm); (b) medium-sized bristles (min-max: 0.5–1.5 mm) covering the whole abdomen and thorax; (c) long thick bristles (min-max: 0.5–3.0 mm) found on the abdomen and the scutellum (Fig 7). Differences in ornamentation also occur between trichomes and bristles. The small trichomes might mimic the small bristles of the fly, whereas the bristle-like trichomes presumably mimic the medium-sized bristles of the fly. Structures that mimic the long bristles of the fly could not be found in *Te*. *peruvianus* but are present in other *Telipogon* species (e.g. *Te*. *falcatus* [34]) with setae on the column.

### Light-microscopic analyses of scent-producing organs in flowers

**Fig 8:**
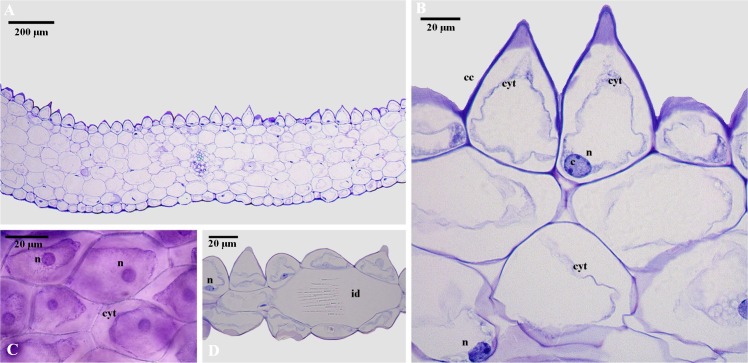
Anatomical details of *Telipogon peruvianus* labellum. (A) Transverse section showing the adaxial and abaxial epidermis and the mesophyll. (B) Conical epidermal cells (cc) on the adaxial epidermis with dense cytoplasm (cyt) and conspicuous nucleus (n). (C) Epidermal cells with dense cytoplasm (cyt). (D) raphide-containing idioblast (id) in the mesophyll. Note the chromocentres (c) inside the nuclei (n). Photographs by C Martel.

We recorded unicellular trichomes and conical cells on the adaxial epidermal surface. Micro-sculpture, as observed by SEM, revealed that they were corrugations of the cuticle, whereas the cell wall was smooth (Fig 8). Trichomes presented a prominent nucleus and dense cytoplasm and contained abundant starch grains. The conical epidermal cells also presented dense cytoplasm and a prominent nucleus (mean ± SD: 14.1 ± 2.2 μm diameter, n = 10) with conspicuous chromocentres (Fig 8). In contrast, the epidermis of the abaxial side was relatively flat and did not exhibit trichomes or papillate cells. The mesophyll was compact, with small intercellular spaces, and possessed large isodiametric cytoplasm-rich cells, also containing vesicles and a large nucleus (mean ± SD: 11.8 ± 2 μm diameter, n = 10) with many chromocentres (Fig 8). Starch grains were also frequently observed in almost all the cells of the mesophyll. Raphide-containing idioblasts were scattered throughout the mesophyll. Neutral red stained the labellum and petals, especially the central region of the flower, corresponding with the white area flanked by the dark red veins (see Fig 1). However, metabolic activity was recorded over the whole corolla, the latter being responsible for scent emission.

### Chemical analysis

**Fig 9:**
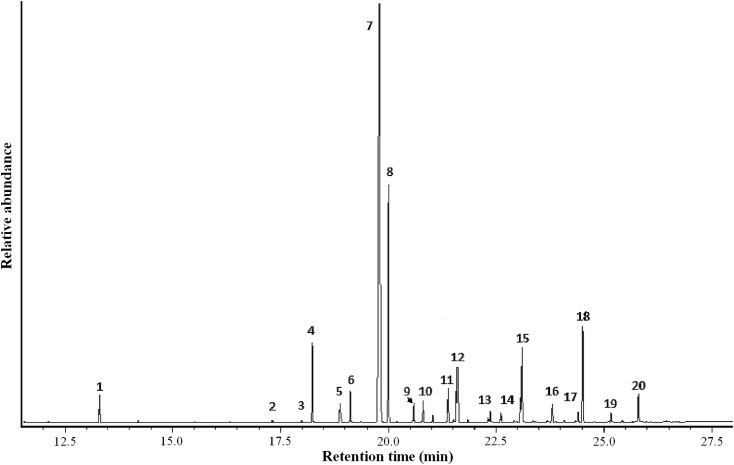
Gas chromatogram of the labellum extract of *Telipogon peruvianus*. Numbered peaks as in Table 2.

In flower extracts of *Te*. *peruvianus*, we identified 23 chemical compounds, 12 alkanes, 10 alkenes and 1 aldehyde (Fig 9). Thus, the floral odour was predominantly composed of saturated and unsaturated hydrocarbons (Table 2). Alkanes and alkenes showed a chain length between 20 and 30 carbons. By far the most abundant compound was (Z)-9-tricosene, whose relative concentrations were up to 60% of the total floral scent extracts.

**Table 2:** Chemical compounds identified by GC-MS in the labellum extract of *Telipogon peruvianus*.

Peak numbers	Chemical compound	Retention index (KI)	Relative proportion (%)
**1**	Tetradecanal	1617	1.21
**2**	Eicosane	2000	0.12
**3**	(Z)-9-heneicosene	2073	0.13
**4**	Heneicosane	2100	3.67
**5**	(Z)-8/(Z)-9-docosene	2174	1.26
**6**	Docosane	2200	1.48
**7**	(Z)-8/(Z)-9-tricosene	2278	60.49
**8**	Tricosane	2300	11.74
**9**	(Z)-8/(Z)-9-tetracosene	2374	1.06
**10**	Tetracosane	2400	1.07
**11**	(Z)-9-pentacosene	2475	1.70
**12**	Pentacosane	2500	2.66
**13**	Hexacosane	2600	0.60
**14**	(Z)-11-heptacosene	2665	0.16
**15**	Heptacosane	2700	3.98
**16**	Octocosane	2800	1.06
**17**	(Z)-7-nonacosene	2885	0.53
**18**	Nonacosane	2900	5.01
**19**	Triacontane	3000	0.51
**20**	Hentriacontane	3100	1.56

Peak numbers refer to Fig 9

## Discussion

Although the genus *Telipogon* was suggested to be sexually deceptive more than 50 years ago [36] and although recurrently asserted since then as being pollinated by sexually aroused male tachinids (e.g. [25, 33, 35, 37–40, 43]), here we confirm, for the first time, that pollination by sexual deception indeed occurs in *Telipogon* and show that *Te*. *peruvianus* flowers are pollinated by tachinid males. Thus, our study represents the first confirmed case of a sexually deceptive pollination system in the Oncidiinae by exclusive pre-copulatory behaviour involving tachinid flies.

In contrast to pollinator behaviour in other sexually deceptive orchids described to date, the males do not show pseudocopulatory behaviour on *Te*. *peruvianus* flowers. We have shown that the pollinia become attached to the legs of the male flies during their attempts to touch and grasp the column and callus of flowers. This behaviour is interpreted as pre-copulatory behaviour, since a similar event has been observed in *Eudejeania subalpina* males before landing on females (Martel pers. obs.) and in the tachinid species *Eucelatoria bryani* [55]. Males of *E*. *subalpina* first touch and then grasp the abdomen and thorax of receptive females that are resting on flowers or leaves and try to make them drop down to lower leaves or to the ground; when this occurs, they move behind the female and copulate (Martel pers. obs.). In *Eucelatoria bryani* touching and grasping are the initial steps of pre-mating behaviour. Unfortunately, studies on the mating behaviour of tachinids are almost completely missing and, therefore, further comparisons with other tachinids are not possible. However, males of *E*. aff. *browni* have developed the same behaviour in the presence of females and flowers. This is a clear indication that males perceived flowers as females, and that *Telipogon peruvianus* flowers use sexual deception but elicit only the first steps of copulatory behaviour in *E*. aff. *browni* males. Although other sexually deceptive orchids have been described in which the removal of pollinia and pollination does not involve pseudocopulation (e.g. [56]) or includes both pre-copulatory and pseudocopulatory behaviour, none of them is associated with pre-copulatory behaviour only [23]. Therefore, to the best of our knowledge, this is the first case of pollination in sexually deceptive orchids involving only pre-copulatory behaviour without further copulation attempts. However, we do not rule out that pseudocopulation events occur on flowers of other *Telipogon* species. We have three assumptions that possibly explain the lack of the development of pseudocopulatory behaviour in *E*. aff. *browni* males: (a) essential tactile cues are missing in the orchid flowers as differences are present in the macro- and micro-structure between *Te*. *peruvianus* flowers (the mimic) and female flies (the model); (b) males are unable to move the pseudo-female away from the flower and therefore do not proceed to try to copulate; (c) odour cues that stimulate pseudocopulatory behaviour are missing in *Te*. *peruvianus* flowers. These assumptions are not exclusive and can occur together. The first assumption is supported by our results of pollinator behaviour in the presence of female dummies and flowers, in which the flower morphology is not perceived as being similar as the female morphology, since males tried to copulate with the dummies but not with the flowers. The third assumption might not be supported by the comparative results but, as the number of pseudocopulations on female dummies was extremely low, the absence of some odour cues cannot be dismissed. As is already known, macro-structure is important for the stimulation of pseudocopulation in some sexually deceptive orchids [57] and a combination of odour, visual and tactile cues is used for mating in some Diptera, e.g. *Drosophila* [58]. Although odour cues play a key role triggering the pseudocopulatory behaviour by pollinators on flowers [22, 59–61], floral morphology, in the presence of identical odour cues, may influence the frequency and duration of the pseudocopulatory behaviour by pollinators [57]. Therefore, a combination of both odour and morphological cues is highly likely to play a role in the absence of pseudocopulatory behaviour of male pollinators. Manipulative experiments are needed in *Telipogon* flowers to identify the importance of odour and morphological cues in this mimicry and to understand the way that pollinators perceive both the model and mimic.

**Fig 10:**
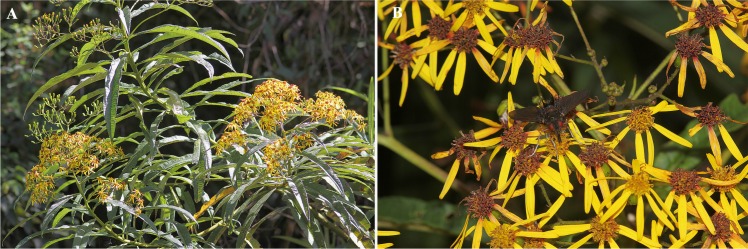
*Dendrophorbium longilinguae* (Asteraceae). (A) Inflorescences of a shrub of *Dendrophorbium longilinguae* (Asteraceae). (B) Groups of several capitules hosting a *Eudejeania* aff. *browni* individual. Photographs by C Martel (left) and M Ayasse (right).

In the sexually deceptive orchids studied so far, the flowers attract males for pollination by mimicking visual cues and sex pheromones of the females of their pollinators [22, 59–63]. Flowers of *Te*. *peruvianus* (and many other *Telipogon* species) present colourful and showy flowers, a common cue in some sexually deceptive species (but see [64–65]). The yellow corolla might increase the visual contrast between the simulated female and the background, with the dark red lines at the labellum bottom possibly acting as ‘landing guides’. The yellow corolla might also mimic the perianth of flowers in which females wait for males. Although several plant species with a yellow corolla occur in the study area, we have only seen males chasing other tachinids and females of *E*. aff. *browni* sitting on inflorescences of *D*. *longilinguae*. Furthermore, both *Te*. *peruvianus* and *D*. *longilinguae* preferentially grow on the margins of the forests, coinciding with the areas of male tachinid routes. Although plants of *Te*. *peruvianus* do not always occur together with *D*. *longilinguae* trees, *Te*. *peruvianus* has been recorded to grow on the trunks of *D*. *longilinguae*. Therefore, we can reasonably hypothesize that *Te*. *peruvianus* flowers mimic *E*. aff. *browni* females sitting on the inflorescence of *D*. *longilinguae*. If so, this would represent a new combined pollination mechanism of rendez-vous attraction and sexual deception in which the mimic imitates two models, the ligulate flowers of the host (*D*. *longilinguae*) and the female tachinid itself (Fig 10). Thus, *Telipogon* has probably developed a system of multifarious floral mimicry during its evolution. This may explain why some *Telipogon* species, and specifically *Te*. *peruvianus*, present nearly actinomorphic flowers.

*Telipogon peruvianus* flowers seem to show an imperfect morphological mimicry as the flower parts and diverse bristle sizes differ between flowers and *E*. aff. *browni* females. Nevertheless, this imperfect mimicry might still be enough to attract and cheat their male pollinators successfully. Therefore, we cannot exclude that the morphological structures of *Te*. *peruvianus* flowers such as the trichomes and papillate cells represent tactile cues in order to stimulate the pre-mating behaviour of males. This is supported by the finding that only a dummy triggered pseudocopulatory behaviour by males, and not flowers. However, further experimental work is needed to evaluate whether males perceive tactilely similarly flowers and females. *Telipogon* flowers present some characteristics of the typical insectiform floral structures present in sexually deceptive orchids such as the presence of hairs and pronounced structures and the dull colours at the flower centre [24]; however, *Telipogon* has other characteristics that are not usual in sexually deceptive orchids such as petals as large as the labellum, a slight dimorphism between the petal and labellum and relatively large flowers. Other sexually deceptive species, such as many *Ophrys* and *Chiloglottis* species, are clearly insectiform and show only a physical mimicry to the females of their pollinators [12, 15, 57, 59, 63]. Furthermore, the presence of only the olfactory stimuli is enough to trigger the mating behaviour of pollinators in the absence of tactile stimuli as shown in *Chiloglottis* and *Drakaea* [22, 61].

Visual stimuli have been suggested as being the most important cues in attracting the pollinators of *Telipogon*, with olfactory stimuli not playing any role. However, contrary to previous reports (e.g. [37, 39, 43]), we show that *Telipogon* flowers are not scentless but release aliphatic compounds such as alkanes and alkenes. We have identified petals and labella as being the source of floral scent production and emission, since they display a conical epidermis, starch deposits, cells with large nuclei, dense cytoplasm and prominent chromocentres and grooves on the epidermis surface [50, 66–68]. In several orchids (e.g. *Chiloglottis*, *Drakaea* and *Ophrys*), odour is the most important cue for attracting pollinators and for stimulating copulatory behaviour on the labellum [11, 12, 22, 60–62, 69]. Alkanes and alkenes are important semiochemicals and often play a role as pheromones in insects [70–72]. Alkanes and alkenes are common in *Ophrys*, especially in *Andrena*-pollinated species, and are known for being responsible for triggering copulation attempts in bee males [32, 60, 69, 73–75]. Alkenes also occur in dipteran pheromones and some fly species even use alkenes as major sex pheromone components such as tricosene and pentacosene in *Drosophila*, *Musca* and *Fannia* [58, 71, 76–79]. Although alkanes are rather common substances in floral bouquets; alkenes have been reported to be rare, but when present they are usually associated with specialized pollination systems involving males such as sexual deception [75]. Therefore, the occurrence of both alkanes and alkenes in flowers of *Te*. *peruvianus* and their known function in some Diptera suggest a role of those chemical compounds as a sexual pheromone in tachinid flies. However, without further behavioural experiments (see [80]), the role of the floral scents in *Telipogon* remains speculative.

As in many other sexually deceptive orchids, ethological isolation barriers [2] play a primary role in the highly specific relationship between plants and their pollinators. Usually, only males of one pollinator species are attracted by most of the *Ophrys* species [30], *Chiloglottis* [29] and *Drakaea* [22]. Three *Eudejeania* species were recorded as being attracted to *Te*. *peruvianus* flowers but only one performed pollination. This might be related to ethological isolation barriers, possibly originating from the composition of the floral odour blend (i.e. the olfactory stimuli). Furthermore, the morphology of the flowers also plays a role as a morphological isolation barrier [2] and allows only males of one pollinator species to remove and transfer a pollinarium. In order to do so, the stipe size should fit to the length of the fly leg and the viscidium diameter should accord with the femur diameter.

Plants of *Te*. *peruvianus* only present one or two flowers in anthesis at a time and this might reduce the probability of geitonogamy. Attracted males usually leave one inflorescence after visiting one flower, thus preventing autogamy and allogamy. Therefore, self-pollination is reduced and pollen flow is encouraged [5, 11]. Van der Pijl and Dodson [33] have noted that *Telipogon* pollination is successful, as seed pods are often found. Our observations in P_1_ have confirmed that the reproductive success of *Te*. *peruvianus* is comparable with that previously reported in most rewarding orchids [81]; only *Cryptostylis subulata* has been recorded to achieve a higher success rate (87%) among sexually deceptive orchids [28]. In *Ophrys*, the flower visitation and pollination rate are usually much lower and often fewer than 5% of the plants are visited by a pollinator [82]. Pollination events in sexually deceptive orchids are maximized by their long-lived flowers [10] and the behaviour of males competing for females [27], as occurs in *Te*. *peruvianus* during the first few hours of the day. Differences in the pollination success between the two *Telipogon* patches are explained by pollinator occurrence and not because of actual ineffective attraction. The limited presence of *Eudejeania* flies in P_2_ might be related to differences in vegetation structure and, in particular, the low presence of nectar host plants compared with P_1_ (Martel pers. obs.).

### Concluding remarks and future prospects

Our findings are the first conclusive report of pollination by sexual deception in the genus *Telipogon*, the subtribe Oncidiinae, involving male tachinids. (a) The pollination of *Te*. *peruvianus* flowers is highly specific and is only performed by males of one *Eudejeania* species. (b) Tachinid flies pollinate *Te*. *peruvianus* flowers, but imperfect morphological mimicry is apparent between the flowers and the females of their pollinator. (c) Flower-visiting males show pre-copulatory behaviour but we have not recorded pseudocopulation. (d) The flowers are self-compatible but are pollinator-dependent in order to develop fruits; the pollination success is one of the highest among sexually deceptive plants studied so far and is similar to some rewarding orchids. (e) *Telipogon peruvianus* flowers release floral scents; this observation challenges previous authors who have studied this taxon, although the role of floral scents in pollination success is not yet fully understood. The next step is to identify the stimuli (visual, olfactory or both) that play a function in the attraction of male tachinids to flowers of *Te*. *peruvianus* and related *Telipogon* species. Further chemical analyses and electrophysiological and behavioural tests are presently in progress.

## Supporting Information

S1 Video A visit of a male of *Eudejeania* aff. *browni* to flowers of *Telipogon peruvianus* in slow motion. (WMV)
